# Supplementary material for: Epithelial development of the urinary collecting system in the human embryo
Source: PLoS One. 2024 Apr 10;19(4):e0301778. doi: 10.1371/journal.pone.0301778 (PMC11006188; doi:10.1371/journal.pone.0301778)
Supplement: Attachment — Submitted filename: Response to Reviwers.docx [file pone.0301778.s004.docx]

Response to the Reviwers

Reviewer #1:

1. Lines 97-98: would be better to clarify the criteria used for differentiation of the choroid plexus and what modifications were used

- Explanations were added in Histological findings, Evaluation of the UCS epithelium, urinary duct, and bladder, in the Materials and Methods sections.

Histological evaluation of the differentiation is not known for the human embryonic urothelium and is limited to another type of embryonic epithelium except for that of the choroid plexus [20,21]. The choroid plexus is a special organ that produces cerebrospinal fluid and is responsible for an important biological barrier system, forming an interface between the blood and the cerebrospinal fluid. Considering the urothelium's several functions described above, some physiological similarities in both structures appear evident. Therefore, it seemed interesting to use a similar differentiation criterion to describe epithelial tissue on the urinary tract whilst including its distinctive features.

2. Line 462: 'retrogradely' is not grammatically correct, use 'in a retrograde fashion'

- The phrase in the conclusion was changed as indicated.

3. Line 464-466: Would read better as '. (F) further study focusing on the histological development of urinary tract in fetus would be necessary to complete the chronology of UCS, ureter and bladder histological development from conception to birth.

- The sentence in the conclusion was changed as indicated.

Reviewer #2:

1. First, it is necessary to clearly state in the introduction why this study is important as a solution to the previous problems. Although this is a significant study, it is difficult to understand its purpose.

- Fourth paragraph in Introduction was reworded for that the purpose and significance of this research become much clear.

Although the urinary tract (including the ureter and bladder) is uniformly lined by the urothelium, the process of histological differentiation during the embryonic period is primarily confined to the bladder [2,3,16] and urinary tract [6], with detailed descriptions lacking for the UCS [12]. Because urine secretion initiates during the late embryonic period [17], clarifying the features and timeline of the histological differentiation of the urothelium region by region is essential. Clinically, this could yield data to ascertain the locations of congenital urinary tract defects, potentially facilitating the initiation of a clinical retrospective study on probable causes. Herein, we aimed to demonstrate the differentiation of the UCS epithelium in the human metanephros during the human embryonic period and to evaluate its degree of differentiation compared to that of the ureter and bladder epithelia.

2. Second, there is no explanation of Figs 6 and 7 in the text, and Figs 8 and 9 are not sufficiently explained.

- Sufficient explanation of Figures 6, 7, 8, and 9 was added. Now reader could understand these Figures easily.

3. Furthermore, a detailed explanation is needed as to why the choroid plexus was chosen for this differentiation criterion.

- Explanations were added in Histological findings, Evaluation of the UCS epithelium, urinary duct, and bladder, in the Materials and Methods sections.

Histological evaluation of the differentiation is not known for the human embryonic urothelium and is limited to another type of embryonic epithelium except for that of the choroid plexus [20,21]. The choroid plexus is a special organ that produces cerebrospinal fluid and is responsible for an important biological barrier system, forming an interface between the blood and the cerebrospinal fluid. Considering the urothelium's several functions described above, some physiological similarities in both structures appear evident. Therefore, it seemed interesting to use a similar differentiation criterion to describe epithelial tissue on the urinary tract whilst including its distinctive features.
